# At Home with *Mastomys* and *Rattus*: Human-Rodent Interactions and Potential for Primary Transmission of Lassa Virus in Domestic Spaces

**DOI:** 10.4269/ajtmh.16-0675

**Published:** 2017-04-05

**Authors:** Jesse Bonwitt, Almudena Mari Sáez, Joseph Lamin, Rashid Ansumana, Michael Dawson, Jacob Buanie, Joyce Lamin, Diana Sondufu, Matthias Borchert, Foday Sahr, Elisabeth Fichet-Calvet, Hannah Brown

**Affiliations:** 1 Department of Anthropology, University of Durham, Durham, United Kingdom; 2 Institute of Tropical Medicine and International Health, Charité-Universitätsmedizin Berlin, Berlin, Germany; 3 Mercy Hospital Research Laboratory, Bo, Sierra Leone; 4 Department of Microbiology, College of Medicine and Allied Health Sciences, University of Sierra Leone, Freetown, Sierra Leone; 5 Department of Virology, Bernhard-Nocht Institute of Tropical Medicine, Hamburg, Germany

## Abstract

The multimammate mouse (*Mastomys natalensis*) is the reservoir for Lassa virus (LASV). Zoonotic transmission occurs when humans are directly or indirectly exposed to fluids of the multimammate mouse, such as urine, saliva, and blood. Housing characteristics and domestic organization affect rodent density in and around households and villages, and are likely to be a risk factor for Lassa fever in humans where the reservoir exists. We use semi-structured interviews (*N* = 51), a quantitative survey (*N* = 429), direct observations, and a rodent ecology study to provide new insights into how the organization of domestic spaces brings together humans and rodents and creates pathways for infection in rural settlements in Bo District, Sierra Leone. Rodents were frequently reported inside houses (92.4% of respondents), in which we predominantly trapped *M. natalensis* (57% of trapped rodents) and *Rattus rattus* (38% of trapped rodents). Building design and materials provide hiding and nesting places for rodents and lead to close proximity with humans. Patterns of contact are both unintentional and intentional and research participants reported high levels of contact with rodents (34.2% of respondents) and rodent fluids (52.8% of respondents). Rodents are also perceived as a serious threat to food security. These results present detailed knowledge about how humans live with and come into contact with rodents, including the LASV reservoir. Our results argue for further collaborative research in housing and environmental modification such as ceiling construction, food storage, and sanitation as prevention against zoonotic LASV transmission.

## Introduction

Lassa fever (LF) is a viral zoonotic illness and a significant cause of morbidity and mortality in countries across West Africa, namely Benin, Guinea, Liberia, Nigeria, and Sierra Leone.^1^–^3^ LF is estimated to affect between 250 and 300,000 people and cause between 5,000 and 10,000 fatalities annually across the region,^3^ but many cases are likely to go unreported due to a lack of diagnostic facilities.

The main reservoir for Lassa virus (LASV) is the multimammate mouse, *Mastomys natalensis*. Other rodent reservoirs (*Mastomys erythroleucus* and *Hylomyscus pamfi*) have been recently identified^4^ but their relative contribution to human infections is unknown. Transmission from rodents to humans occurs through direct exposure to rodent fluids such as urine, saliva, and blood or indirect exposure via surfaces and foodstuffs contaminated by these fluids.^5^,^6^ Urine may present a particular risk for human infections as *M. natalensis* can shed LASV in urine at any age^7^ and LASV has been shown to be aerosolized under laboratory conditions.^8^ Secondary human-to-human transmission follows contact with human bodily fluids in the household or health-care facilities, and is estimated to occur in 20% of LF cases.^9^ Risk factors for primary (zoonotic) transmission are unclear and possibly linked to housing^10^ and hunting and consumption of rodents.^11^–^13^

No licensed vaccine exits but the antiviral ribavirin can improve prognosis if administered early after symptoms appear. Current recommendations for the prevention of primary transmission focus on reducing rodent abundance in houses and surrounding spaces, improving sanitation (rodent proofing houses and/or stored food), and avoiding direct contact with rodents as occurs during hunting and consumption.^14^ Preventing primary transmission in this way requires detailed knowledge about how humans live with and come into contact with *M. natalensis*.^15^

In West Africa, the prevalence of LASV in *M. natalensis* ranges between 5% and 20%.^16^–^19^ In Upper Guinea, *M. natalensis* comprises between 95% and 98% of rodents captured in houses.^20^ In coastal Guinea, the black rat *Rattus rattus* enters into houses and tends to evict *M. erythroleucus*.^21^ In Sierra Leone, both species are present, with *R. rattus* already recorded in 1972 in Panguma,^22^ and in 1978–1980 in many other localities (J. Krebs in GBIF database; http://www.gbif.org/species). Houses, kitchens, and stores built with mud and wattle provide rodents with increased opportunities to burrow and food stores attract and support rodent populations.^3^,^23^

A conclusive causal link between housing quality and human LASV infection has yet to be determined, the principal difficulty residing in the fact that the existence of other potential risk factors in the domestic environment makes it difficult to disentangle various risks. In a study of refugee camps in Sierra Leone, Bonner and others^10^ found that the presence of rodent burrows, and external hygiene around the house in particular, was directly associated with a history of LF in the household. The presence of rodent burrows in turn was directly associated with housing quality (defined as construction material used and current state of maintenance). In Nigeria, there was no statistical difference between LASV-positive and LASV-negative households with regard to housing quality, but there was an association between housing hygiene (defined as waste disposal and food storage) and a (self-reported) history of LF in the household.^24^ In Sierra Leone, Moses and others^25^ found a correlation between *M. natalensis* trapping success and rodent burrows in the home; however, trapping success was not correlated with wall or roof type, and only weakly with floor construction. Seroprevalence of LASV antibodies was not associated with presence of rodents in households in Guinea.^12^

Nevertheless, housing characteristics that lead to an increased rodent density in and around households and villages are likely to be a risk factor for LF in humans^10^ and warrant further investigations.^26^–^29^ However, there is little information describing the specificities of rodent–human interaction inside homes and facilitators and barriers such as construction methods and domestic organization. This study seeks to address this gap by describing how household organization creates the conditions for contact between humans and rodents and provides insights on how these interactions may form pathways for infection.

## Methods and Materials

We combined qualitative and quantitative surveys to capture a finely grained picture of rodent–human interactions. We place our observations into perspective by presenting results from our rodent ecology survey. Ethical clearance was received from the ethics committee of the Government of Sierra Leone, Charité-Universitätsmedizin, Berlin, and the Royal Veterinary College, London. Written consent was obtained from all participants.

### Study sites.

In Bo District, the Mende form the majority ethnic group (79%) followed by the Temne (7%). Islam (72%) and Christianity (27%) are the two principle religions.^30^ The main economic activities are crop farming, diamond mining, and construction work.^30^ A majority of the population (60%) is rural. Fishing, hunting, and farming (rice, cassava, yam, and sweet potato) serve as means of subsistence or as income generating activities with pineapple, mango, coffee, cacao, and palm oil as main cash crops.^31^

**Figure 1.**
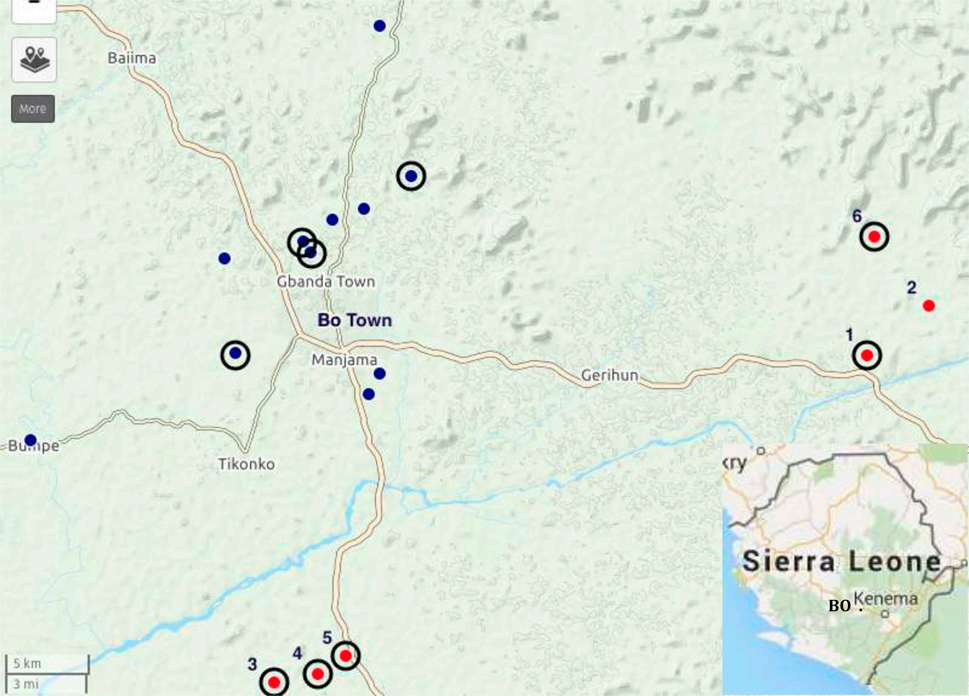
Location of the 17 study sites in the vicinity of Bo Town. Red dots = rodent survey; dots with circles = quantitative survey; all dots = qualitative survey; numbers refer to villages in Table 2 (created with UMAP http://umap.openstreetmap.fr).

We conducted anthropological fieldwork in Bo District (southern province) over a period of 4 months (May–June 2014 and October–December 2015). Rodent ecology investigations took place between April 2014 and February 2015. Making use of the long-standing presence of our local research team in the area since 2010, we identified 17 villages of varying size (500–1,500 inhabitants) and distance from main transport axes (from 4.5 to 40 km from the outskirts of Bo Town) (Figure 1

).

### Anthropological investigations.

#### Qualitative survey.

In all 17 study villages, we applied common methods to collect qualitative data until saturation was achieved: in-depth interviews (IDIs, *N* = 51), spontaneously occurring focus groups discussions (*N* = 4), and observations (over the entire duration of the study period). Potential study participants were identified through our local researchers' previous work in the area and were purposefully selected to achieve representation from various groups (socioeconomic status, profession, religion, ethnicity, age, and sex).

The principal topics included in our interview and observation guides covered contact with rodents and their fluids inside homes, perceptions of rodent behavior and ecology (e.g., feeding, nesting), materials, design and maintenance of dwelling spaces, food security and storage (damage caused by rodents to foodstuffs), types of rodent control measures, and knowledge of LF (transmission routes, symptoms, and prevention strategies). Patterns of contact that occurs during hunting and consumption of rodents were also explored as part of this study but are described in a separate paper.^13^ Assuming that the presence of peri-domestic rodents is related to the physical set up of domestic spaces, we paid particular attention to the construction and spatial organization of houses. Qualitative protocols are usually divided into two phases, which are iterative and complementary: the first one is informed by a literature survey to design the principal lines of research, in our case corresponding to biomedical risk factors for disease transmission (e.g., direct and indirect contact with rodents and their fluids) and factors that affect rodent ecology (e.g., feeding and nesting). The second phase occurs during fieldwork, where the daily preliminary narrative analysis of transcripts and field notes helps adapt the interviews and observations guides to the emergent lines of investigations.

Discussions were carried out in Mende, Krio, or English and facilitated by a translator. Formal discussions were recorded and transcribed. Informal discussions and observations were documented with field notes and photographs. Interviews lasted on average for 1 hour and were conversational and open-ended, treated as occasions for a mutual exchange of information with as much time as possible to informal interactions with the communities to establish trust.

Recordings and field notes were immediately transcribed using MS Word 2011 (Microsoft Corp., Redmond, WA). Individual and village identifiers were removed and coded to ensure anonymity. The transcripts were reviewed using a thematic analysis and segments of interests were color coded according to the topics described earlier. Analysis was done on a daily basis so that questions and observation guides could be refined in an iterative fashion. Reflective notes were made daily, compared with published literature, and regularly shared with the research group.

#### Quantitative survey.

A cross-sectional questionnaire survey was carried out midway during the first fieldwork period (May–June 2014). We purposefully selected nine villages out of the 17 study villages to represent different population sizes and distance from main transport axes. Selection of individuals was carried out according to the World Health Organization Expanded Programme on Immunization Coverage Survey method.^32^ In total, 524 subjects were recruited (see details in Bonwitt and others, 2016). Fifty-seven records were excluded because respondents lived in a major city, 21 because respondents lived in a village other than the study villages, and seven because the village name was not indicated on the questionnaire.

The questions were based on findings from a first set of IDIs and covered all forms of contact with rodents (contact in homes and farms, contact during hunting, butchering, and consumption) as well as food security and knowledge of LF. A total of 55 questions were asked. The answer format relevant to the questions described in this study was either single or multiple choices. Questions were in English and administered by local staff trained to translate the questions in Krio and Mende.

Records with answers stating “unknown” or “don't know” were not included in the analysis for that particular question. The final number of respondents varies according to question because skip logic was used to avoid asking redundant or irrelevant questions based on the respondent's previous answers. Data were collated and analyzed with STATA 13 (StataCorp. 2013; StataCorp LP, College Station, TX) and MS Excel 2011 (Microsoft Corp., Redmond, WA). We estimated proportions of subjects with contact with rodents, control measures, and food security. The adjusted Wald method was used to calculate 95% confidence intervals.

### Rodent survey.

Of 17 villages investigated for this study, six were chosen for rodent sampling (Figure 1). These villages were chosen according to criteria that limit colonization of *R. rattus*, in villages and which could lead to displacement of other rodent species. The criteria included: village population between 500 and 1,000 people, village surrounded by forest or wooded savanna, absence of paved road access to the village, absence of weekly markets, and location within 45 minutes driving distance from Bo Town. The commensal rodents were sampled in April 2014, July 2014, October 2014, and February 2015. Usually, 100 large folding aluminum Sherman traps were set inside houses, kitchen, and stores if separate from the main house, along a transect crossing the village. Two to 12 traps per house (depending on the size of the house) were set during three consecutive nights of each trapping session. In July 2014, the trapping session was reduced because of challenges brought by the Ebola virus disease outbreak. The total trapping effort for the four sessions reached 5,868 trap-nights. Traps were checked each morning, and animals were necropsied in a safe location near the village, according to BSL3 procedures.^33^,^34^ Morphological identification was done in situ by weighing and measuring the animals. As several species of *Mastomys* can live in the area, further molecular identification based on the cytochrome b was done in the laboratory.^35^

## Results

We provide a statistical description of the study participants from all nine villages chosen for the quantitative survey (Table 1).

### Domestic spaces.

The supporting structures of houses in the study villages are built from various materials, including cement brick, earth/clay brick, or from earth/clay and wattle over a supporting skeleton built of wooden poles woven with smaller branches (these latter two structures have a lifespan of several years). Walls are sometimes plastered with cement. Roofs are either made of thatch (from palm trees) that require re-thatching every 1–3 years, or corrugated metal that usually requires little repair over a lifetime. Floors are either dried mud or cemented. Houses and other structures (schools, religious edifices, and place for community meetings) built with cement are rare.

**Figure 2.**
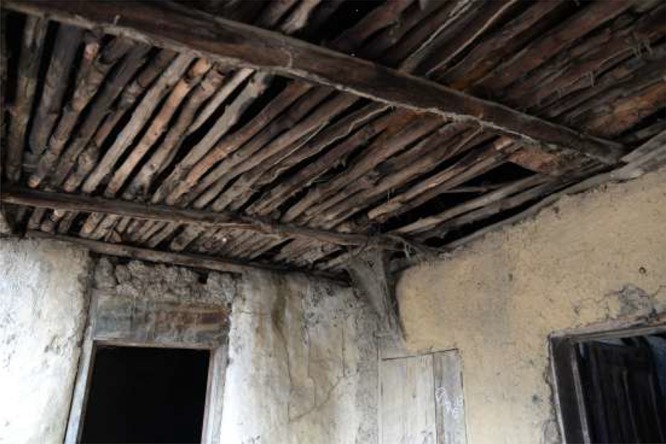
House ceiling made of aligned branches obtained from the forest.

Indoors, ceilings are built to create a lower boundary under the roof and storage for rarely used objects. Ceilings are typically formed by an alignment of dried branches (Figure 2

), which may be covered with mats made from plant fibers. Ceilings made from other materials such as corrugated metal or wood planks are uncommon. Houses generally consist of multiple rooms with a single room serving many purposes: bedroom, storage or, sometimes, for small businesses. Most houses and kitchens have a veranda for cooking and eating, but people also cook indoors during rainy or cold periods. A kitchen consists of an open fire on the ground with three stones supporting the cooking pot. Spilled raw and cooked food is swept aside but not removed at night. Corridors are used for storing various objects such as cooking utensils (mortars, pots) and agricultural products. Cupboards or trunks are rare and possessions (clothes, cooking utensils, and agricultural and fishing equipment) can often be found heaped on the floor, stored in plastic buckets with lids, or hung from the ceiling or walls. Storerooms and corridors are usually devoid of windows. Bedroom windows (without glass) are invariably small, and, in the absence of the owner, shutters are kept closed during the day. The little light that penetrates inside houses does so through cracks in shutters, doors, and holes in the roof. Electricity is nonexistent save for an occasional generator often shared among village members, and the only commonly available light sources in villages are battery-powered torches.

Outdoors, villages have well-trodden earth in areas immediately around and between houses with occasional shrubs or bushes, sometimes interspersed with abandoned and crumbling homes invaded by grasses and shrubs, which are regularly cut to flush out rodents. Latrines, where these exist, are placed at some distance from the house, often at the junction with the bush. Garbage (notably food leftovers and rice husk) is disposed of in pits or more commonly openly thrown on the ground on the outer limit of the village.

Farmhouses serve as simple second homes and are located away from people's main homes close to their agricultural land. They constitute an individual unit of domestic space in the “bush” and are used to facilitate agricultural work (including resting, cooking, and storage). In essence, farmhouses in the bush mirror houses in villages, with similar but simpler and more temporary structures.

### Food stock and cooking uses.

Grains, leguminous crops, and fruit are stored on the floor in covered buckets or large flour bags. Food left over from the evening meal is kept for the following morning. Such food, termed “sleep rice” or “cold rice,” is usually stored overnight in covered pots and eaten for breakfast. Wealthier people have better quality containers for storing both cooked and raw food (e.g., pots with fitting lids, wooden trunks for food, and other possessions). Bowls and utensils are not always washed immediately after use because of the lack of running water and lighting, especially after the evening meal. Younger female household members are traditionally expected to wash these in the morning. Grain (principally rice) is stored on ceiling rafters, inside the home, or in designated grain stores outside the main dwelling area made of thatch, which sometimes double as kitchens. For subsistence farmers, the stored rice harvest is meant to last the whole year for household consumption, sale, gifts, and ceremony contributions, and to provide the next year's seeds. Storing foods indoors, in particular rice, was reported to be a major source of attraction to rodents.

### Contact with rats.

Small- to medium-sized rodents are collectively termed “rats” in English (“arata” in Krio), a terminology that we continue in the result section when referring to the word “rat.” Our research participants reported both unintentional (and generally undesired) forms of rodent–human contact as well as intentional contact with *M. natalensis* and other rodent species. These forms of interaction sometimes involved direct or indirect contact with rodent urine, feces, or blood. Our rodent survey in six villages during a 1-year period showed that *M. natalensis* shared the domestic space (defined here as houses within the study villages) with *R. rattus* (Table 2).

**Figure 3.**
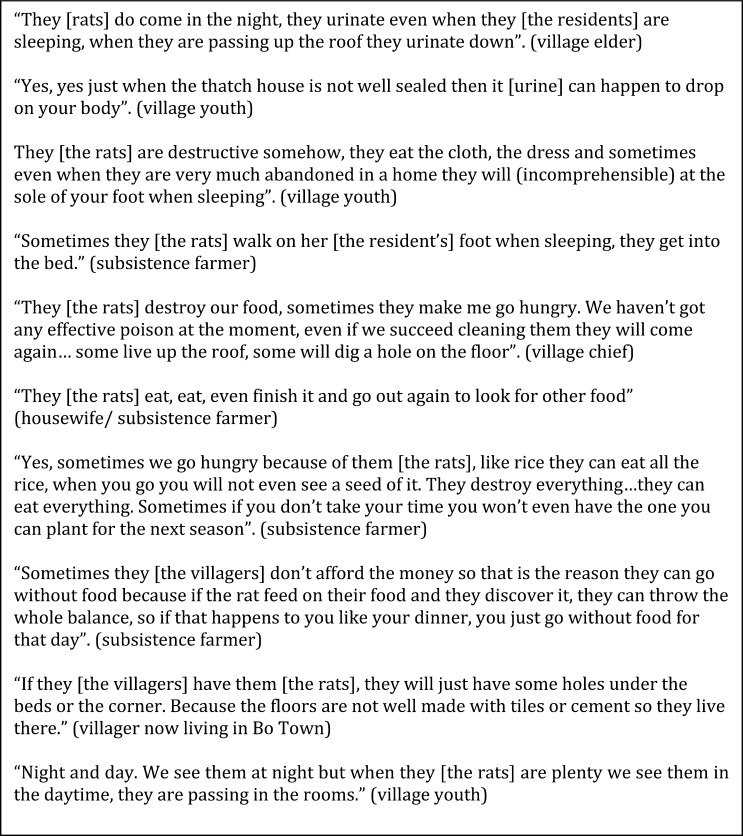
Reported interactions between humans and rats (excerpts from qualitative survey).

Our quantitative survey indicates that a large portion of people have contact with live rats (34.2%, 150/439) or rat urine (52.8%, 232/439) (Table 3). In the morning, evidence of nocturnal activity was found through the presence of feces and rice husks around dishes and grain stores. Another undesirable form of unintentional direct contact occurred at night, with people describing having the soles of their feet occasionally nibbled by rats during their sleep, which was considered an omen of death in the family by some. The most frequently discussed form of unintentional contact with fluids from rats occurred at night, when the hut becomes alive with activity indicated by the incessant sounds of soft-footed movement. Showing little respect for their host, rats urinate down from the interspersed rafters onto the household members. Even though this does not necessarily interrupt the residents' sleep, the pungent smell of rat urine and yellow stains in the morning served as a reminder of the nightly visit. Informants discussed this casually as an unpleasant event but part of daily life (Figure 3

).

Informants reported that intentional contact between humans and rats within villages was mostly restricted to children. This was corroborated with observational data. It is common for children to keep young animals of various species, including small rats, as pets. Neonate rats are caught when a nest is discovered, and children described playing with older rats when they are found “drunk” with poison.

Attempts to control rats inside homes are common, with a majority of informants (85.0%, 373/439) using some form of rat control including poison (76.8%, 337/439), cats (28.5%, 125/439), and traps (23.0%, 101/439) (Table 3). Trapping and poisoning are done in a reactive rather than preventive fashion and is mainly undertaken through individual rather than collective initiative. Other measures against rats include storing prepared and raw food in covered pans with lids. People of all age and gender will also opportunistically kill rats using whatever is at hand (e.g., sticks, stones, machetes). For example, rat abundance is considered so high that dismantling old thatch roofs during repairs is considered an opportunity to kill rats as they are dislodged and people will prepare to catch rats that flee on these occasions.

### Rats as a threat to food security.

A frequently recurrent theme discussed spontaneously by informants was the material damage caused by rats in homes and on farms. Informants overwhelmingly reported that rats ate leftover food, destroyed grain stores and even other possessions such as clothes, bags, and bank notes (Figure 3). It is common to see container bags eaten through and harvests can be completely lost if the damage is not spotted early enough. In this respect, rats are considered voracious animals. Many people regularly reported rats contaminating food that could not be stored safely and the need to make the difficult decision of throwing cooked food away, although some informants claimed that they could not afford to do so, or they would forfeit the next meal. In addition, rats destroy grains that are needed to plant the next year's crop. Table 3 provides further evidence of the widespread negative impact of rats with 90.0% (395/439) and 85.0% (373/439) of individuals, respectively, reporting damage to food stores and crop plantations. Steps are taken to minimize damage caused by rats, such as hanging bags from rafters, but even these are not always effective.

## Discussion

Overall, there was consensus between the quantitative and quantitative results regarding contact with rodent and rodent control measures: contact with rodents and their body fluids was found to be widespread, and damage to food stores was significant However, study participants may have over reported the impact of rodents in the hope of receiving benefits such as interventions to decrease rodent abundance or improve food security.

### Building use, materials and design, and peri-domestic rodents.

In Bo District (excluding urban Bo City), most houses are thatched (20.9%) with mud/mud and wattle walls (77%) and earth floors (59.2%). A majority of these are deemed to require minor (66.1%) or major repairs (20.8%).^30^ These natural building materials are obtained from the surrounding bush (bamboo, wood, thatch), are friable, and provide opportunities for burrowing. The clutter lining walls and floors allow for furtive movements suitable to rodent behaviors and can provide habitats for rodents without the need for burrows.

The high abundance of rodents within homes reported by household members (92.4%, 404/437) is in line with previous surveys in the eastern province of Sierra Leone (86%)^36^ and is likely to be linked to building materials and modes of domestic organization in the region. One study in urban Sao Paulo, Brazil, found that environmental characteristics similar to the ones described in this study were strongly correlated with rodent infestation. The odds of urban premises to be infested by rodents was 4.5 times higher when there were access facilities (defined by building structure or sewage), 3.2 times higher with harborage sources (dense bush, derelict materials, ceiling, and wall cracks), and 1.6 times higher with the presence of various food sources.^37^ Similar environmental determinants for rodent infestation (based on observations by villagers) were observed in villages in Lao People's Democratic Republic, notably housing structure (open ceilings), presence of rubble, and access to food.^38^ In hindsight, it would have been worthwhile for our survey to include housing infrastructure (such as wall, ceiling, and roof materials) and a measure of the status of repair to determine a possible correlation with rodent infestation.

Ecology studies suggest that rodent abundance in houses doubles during the dry season indoors, possibly as a result of restricted food supply outdoors and increase food supply indoors.^19^ This may be due to storing harvests on ceilings that may attract rodents, whose movements are facilitated by roof and ceiling constructions and where it is harder to instigate rodent control measures.

In the bush, many daily activities such as cooking, resting, and certain agricultural activities occur in farmhouses. Their structure (e.g., thatch roofs and grain stores) echoes those of houses and encourages commensality between rodents and humans similar to those described in villages. However, the site of these rodent–human interactions occurs in different ecotones (farmhouse/agricultural land/forest), where the species richness may differ from those in villages. Further, the location determines how humans perceive rodents, and in contrast to villages, contact with rodents outside of villages is often intentional and motivated by various factors related to rodents as agricultural pests and a source of food.^13^

### Direct risks for zoonotic transmission.

Research participants reported high levels of contact with rodents and rodent fluids, particularly at nighttime when levels of rodent activity in houses were highest and when rodents moved around domestic spaces in close proximity to humans. The permanently dark conditions created indoors probably extend the crepuscular activity of *M. natalensis*^28^,^39^ and provide increased opportunities for environmental contamination. Further, the absence of ultraviolet light indoors may also prolong virus survival on surfaces^40^ contaminated by rodents.

Our quantitative survey indicates that a large portion of people report contact with live rodents or rodent urine, the latter being facilitated by the roof and ceiling structure that favor rodent activity. We identify this as a possible transmission route given that infected rodents secrete arenaviruses and Morogoro virus in urine and feces,^7^,^41^,^42^ and that LASV has been shown to be aerosolized under laboratory conditions.^8^ We cannot conclude that the respondents of the quantitative survey who reported exposure to urine were exposed specifically to urine from ceilings because the questionnaire did not specify the urine source. However, we can infer from our qualitative data that urine contamination from ceilings is widespread and common. Further, we did not specifically trap in ceilings so we cannot conclude that *M. natalensis* is the specific culprit of ceiling urination. Our rodent ecology data show that the two main species cohabiting with people are *R. rattus* (38% of rodents trapped) and *M. natalensis* (57% of rodents trapped). Colonization of ceilings is more likely due to *R. rattus* (commonly termed the roof rat), which is the most agile climber among the species caught during the rodent survey.^43^–^45^ Colonization of ceilings by this species is especially likely in villages in proximity to Bo Town (nine out of 17 villages for our anthropological investigation) because *R. rattus* is more abundant than *M. natalensis* near urban areas and major transport axes.^46^ Future research could determine the spatial distribution of different species within houses. For example, preferential colonization of ceilings could pose a risk for LF and other urine-borne zoonotic diseases, whereas ground floor colonization could pose a risk through food contamination. Finally, our data might underestimate the abundance of *R. rattus* because we used large folding aluminum Sherman traps. These traps are smaller than the full length (rostrum to tail) of an adult *R. rattus* and might have discouraged them from entering our traps.

We previously reported that rodents found outside of villages (“bush rats”) are hunted for food but that rodents found in villages (“town rats”) are not eaten because of their association with disease.^13^ Herein, we describe forms of contact with rodents found in villages that are generally unintentional and unwanted. However, many adult informants have been unwilling to admit to intentional contact with rodents (e.g., for consumption) within villages. Although our data suggest that most people differentiate between these two categories of rodents for the purpose of consumption, there is likely to be a degree of overlap depending on personal degrees of tolerance for eating rodents that are deemed to carry diseases. Intentional contact with rodents within villages was described as being restricted to children, which places them at risk through bites and contact with fluids of adults and neonates rodents, which can shed LASV at any age.^47^

Contact with rodents in and around houses was frequent, intimate, generally undesired, and possibly associated with specific features of the structure of dwellings and the organization of domestic space. Thus, the behavior of rodents and humans and ways in which they overlap have relevance for the eco-epidemiology of LF and other rodent-borne diseases (e.g., plague, hemorrhagic fever with renal syndrome, relapsing fever, rickettsiosis, toxoplasmosis), including those transmitted through urine (e.g., leptospirosis).^48^ This is of particular importance considering the role of rodents in emerging infectious diseases^49^,^50^ and the recent discovery of new reservoirs for LASV^4^ that have a different ecology to *M. natalensis*.

### Aspects of rodent control.

The majority of study participants used some forms of rodent control. Trapping and poisoning are done in a reactive rather than preventive fashion and mainly undertaken through individual rather than collective initiative. This is likely to have minimal effects due to rapid recolonization as opposed to preventive and coordinated control at household, compound, or village level.^51^–^53^ The frequent requests for help or advice on rodent control received during fieldwork was an indication of the overwhelmingly pernicious influence rodents had on everyday life and the difficulty of controlling them. Rodent damage contributes significantly to food wastage poses a threat to food security, which is of particular concern in a country where more than half of the population lives below the poverty line^54^ and malnutrition is the second leading cause of death.^31^

Reducing the frequency and intensity of contact between *M. natalensis* and humans remains the sole prevention measure against LF infection. Our research suggests that a different rationale toward rodent prevention is needed depending on spatial locations. In swidden and forests, contact with rodents is often motivated or intended, notably during hunting and consumption of rodents^13^; prevention strategies are best focused on sensitization. In domestic spaces, however, contact with rodents are usually unintended or undesired; prevention strategies are best focused on improving rodent control measures including through building materials, structures, and maintenance.

It is unlikely that rodent control alone is sufficient to reduce LF incidence.^28^,^55^ There is little published evidence on the efficacy of rodent proofing of houses in tropical settings. Two studies in rural United States suggest that relatively inexpensive rodent proofing measures can decrease the frequency and intensity of rodent activity inside houses.^56^,^57^ Our observations suggest possibilities for additional targeted forms of environmental modification that could improve the reduction of rodent abundance and the frequency of contact with humans. These include improving ceiling construction, doors, windows, junctions between walls and roofs, and removing sources of attraction by improving methods of food storage.

Further, people should be encouraged to avoid direct contact that occurs when dead or dying rodents are removed from the house following trapping or poisoning. In this instance, communities do not consider contact with dead rodents a risky activity, yet disposing of dead rodents may serve as an additional risk for LF exposure, which needs to be taken into account by intervention strategies favoring rodent control.

## Conclusions

Domestic settings are hypothesized to be important sites for instances of primary transmission.^19^,^39^ This study opens the black box of zoonotic transmission within domestic spaces and provides a description of the frequent and intense patterns of rodent–human interactions, drawing on data collected in rural settlements in Bo District, Sierra Leone. Our data show the value of social scientific and observational methodologies for gaining detailed understanding of potential pathways of zoonotic transmission. At the root of rodent–human interactions lies structural poverty—poor housing infrastructure and lack of basic amenities encourage colonization by rodents and increase the frequency and intensity of rodent–human contact.

We support the call for further collaborative research in housing improvement (building materials and design) and environmental modification to make houses less attractive to rodents as tools against LF.^27^ These are likely to have high levels of acceptance because they address the concerns of community members. Such interventions can be further justified as they are likely to impact other rodent-borne and poverty-related diseases while at the same time contributing to food security.

## Figures and Tables

**Table 1:** Sociodemographic characteristics of study participants (quantitative survey)

Characteristics	No. of recruited subjects, *n* (%)
Overall	439 (100)
Gender	
Female	240 (54.7)
Male	199 (45.3)
Age group (years)	
5–14	67 (15.3)
15–24	92 (21.0)
25–39	140 (31.9)
40 or above	140 (31.9)
Educational level	
None	149 (33.9)
Primary	116 (26.4)
Secondary or above	74 (16.9)
Other*	100 (22.8)
Ethnicity	
Mende	393 (89.5)
Other	46 (10.5)
Religion	
Muslim	343 (78.1)
Christian	94 (21.4)

* Usually refers to Koranic schooling.

**Table 2:** Distribution of commensal small mammals in six villages in Bo District (total of four trapping sessions)

Species	Village 1	Village 2	Village 3	Village 4	Village 5	Village 6	Total
*Crocidura* spp.			1	2		1	4
*Mastomys erythroleucus*	1		1	1	1	1	5
*Mastomys natalensis*	30	57	3	41	15	11	157
*Praomys rostratus*	2			4			6
*Rattus rattus*	23	10	18	27	23	4	105
Total	56	67	23	75	39	17	277
% *M. natalensis*	54	85	13	55	38	65	57

**Table 3:** Contact with, control of, and consequences of interaction with rats (quantitative survey)

	No. of recruited subjects (*n*/*N*)	Estimated proportion (95% CI)
Direct and indirect contact with rats		
Presence of rats in or around the house	404/437	92.4 (89.5–94.6)
Contact of rats with food	393/439	89.5 (86.2–92.1)
Contact with rat urine or feces during the day or at night	232/439	52.8 (48.1–57.6)
Touch live rats	150/439	34.2 (29.8–38.8)
Control measures		
Rat control	373/439	85.0 (81.2–88.1)
Poison	337/439	76.8 (72.5–80.6)
Cat	125/439	28.5 (24.4–33.0)
Traps	101/439	23.0 (19.2–27.3)
Other	54/439	12.3 (9.5–15.8)
Food security		
Food destruction by rats	395/439	90.0 (86.7–92.5)
Crop destruction by rats	373/439	85.0 (81.2–88.1)
Goes hungry because of food/crop destruction by rats	180/405	44.4 (39.6–49.4)

CI = confidence interval.

## References

[1] Fichet-Calvet E, Rogers DJ (2009). Risk maps of Lassa fever in West Africa. PLoS Negl Trop Dis.

[2] Gunther S, Lenz O (2004). Lassa virus. Crit Rev Clin Lab Sci.

[3] McCormick JB, Fisher-Hoch SP (2002). Lassa fever. Curr Top Microbiol Immunol.

[4] Olayemi A, Cadar D, Magassouba N, Obadare A, Kourouma F, Oyeyiola A, Fasogbon S, Igbokwe J, Rieger T, Bockholt S, Jerome H, Schmidt-Chanasit J, Garigliany M, Lorenzen S, Igbahenah F, Fichet JN, Ortsega D, Omilabu S, Gunther S, Fichet-Calvet E (2016). New hosts of the Lassa virus. Sci Rep.

[5] McCormick JB, Saluzzo JF, Dodet B (1999). Lassa fever. Emergence and Control of Rodent-borne Viral Diseases.

[6] Ogbu O, Ajuluchukwu E, Uneke CJ (2007). Lassa fever in West African sub-region: an overview. J Vector Borne Dis.

[7] Walker DH, Wulff H, Lange JV, Murphy FA (1975). Comparative pathology of Lassa virus infection in moneys, guinea-pigs, and *Mastomys natalensis*. Bull World Health Organ.

[8] Stephenson EH, Larson EW, Dominik JW (1984). Effect of environmental factors on aerosol-induced Lassa virus infection. J Med Virol.

[9] Lo Iacono G, Cunningham AA, Fichet-Calvet E, Garry RF, Grant DS, Khan SH, Leach M, Moses LM, Schieffelin JS, Shaffer JG, Webb CT, Wood JL (2015). Using modelling to disentangle the relative contributions of zoonotic and anthroponotic transmission: the case of Lassa fever. PLoS Negl Trop Dis.

[10] Bonner PC, Schmidt WP, Belmain SR, Oshin B, Baglole D, Borchert M (2007). Poor housing quality increases risk of rodent infestation and Lassa fever in refugee camps of Sierra Leone. Am J Trop Med Hyg.

[11] Ter Meulen J, Lukashevich I, Sidibe K, Inapogui A, Marx M, Dorlemann A, Yansane ML, Koulemou K, Chang-Claude J, Schmitz H (1996). Hunting of peridomestic rodents and consumption of their meat as possible risk factors for rodent-to-human transmission of Lassa virus in the Republic of Guinea. Am J Trop Med Hyg.

[12] Kernéis S, Koivogui L, Magassouba N, Koulemou K, Lewis R, Aplogan A, Grais RF, Guerin PJ, Fichet-Calvet E (2009). Prevalence and risk factors of Lassa seropositivity in inhabitants of the forest region of Guinea: a cross-sectional study. PLoS Negl Trop Dis.

[13] Bonwitt J, Kelly AH, Ansumana R, Agbla S, Sahr F, Saez AM, Borchert M, Kock R, Fichet-Calvet E (2016). Rat-atouille: a mixed method study to characterize rodent hunting and consumption in the context of Lassa fever. EcoHealth.

[14] Center for Disease Control and Prevention (2014). Lassa Fever Prevention.

[15] Brown H, Kelly AH, Mari Saez A, Fichet-Calvet E, Ansumana R, Bonwitt J, Magassouba N, Sahr F, Borchert M (2015). Extending the “social”: anthropological contributions to the study of viral haemorrhagic fevers. PLoS Negl Trop Dis.

[16] Lecompte E, Fichet-Calvet E, Daffis S, Koulemou K, Sylla O, Kourouma F, Dore A, Soropogui B, Aniskin V, Allali B, Kouassi Kan S, Lalis A, Koivogui L, Gunther S, Denys C, ter Meulen J (2006). *Mastomys natalensis* and Lassa fever, West Africa. Emerg Infect Dis.

[17] Leski TA, Stockelman MG, Moses LM, Park M, Stenger DA, Ansumana R, Bausch DG, Lin B (2015). Sequence variability and geographic distribution of Lassa virus, Sierra Leone. Emerg Infect Dis.

[18] Safronetz D, Sogoba N, Lopez JE, Maiga O, Dahlstrom E, Zivcec M, Feldmann F, Haddock E, Fischer RJ, Anderson JM, Munster VJ, Branco L, Garry R, Porcella SF, Schwan TG, Feldmann H (2013). Geographic distribution and genetic characterization of Lassa virus in sub-Saharan Mali. PLoS Negl Trop Dis.

[19] Fichet-Calvet E, Lecompte E, Koivogui L, Soropogui B, Dore A, Kourouma F, Sylla O, Daffis S, Koulemou K, Ter Meulen J (2007). Fluctuation of abundance and Lassa virus prevalence in *Mastomys natalensis* in Guinea, West Africa. Vector Borne Zoonotic Dis.

[20] Fichet-Calvet E, Audenaert L, Barrière P, Verheyen E (2009). Diversity, dynamics and reproduction in a community of small mammals in Upper Guinea, with emphasis on pygmy mice ecology. Afr J Ecol.

[21] Fichet-Calvet E, Lecompte E, Veyrunes F, Barriere P, Nicolas V, Koulemou K (2009). Diversity and dynamics in a community of small mammals in coastal Guinea, West Africa. Belg J Zool.

[22] Monath TP, Newhouse VF, Kemp GE, Setzer HW, Cacciapuoti A (1974). Lassa virus isolation from *Mastomys natalensis* rodents during an epidemic in Sierra Leone. Science.

[23] Isaacson M (1975). The ecology of *Praomys* (Mastomys) *natalensis* in southern Africa. Bull World Health Organ.

[24] Ochei O, Abjejegah C, Okoh EC, Abah SO (2014). Housing factors and transmission of Lassa fever in a rural area of south Nigeria. General Health and Medical Sciences.

[25] Moses LM, Kargbo K, Koninga J, Robert W, Lungay V, Fionnie R, Kanneh L, Bangura J, Garry RF, Bausch DG (2009). Household predictors of abundance of the Lassa virus reservoir, *Mastomys natalensis*, in the Eastern Province of Sierra Leone.

[26] Demby AH, Inapogui A, Kargbo K, Koninga J, Kourouma K, Kanu J, Coulibaly M, Wagoner KD, Ksiazek TG, Peters CJ, Rollin PE, Bausch DG (2001). Lassa fever in Guinea: II. Distribution and prevalence of Lassa virus infection in small mammals. Vector Borne Zoonotic Dis.

[27] Kelly JD, Barrie MB, Ross RA, Temple BA, Moses LM, Bausch DG (2013). Housing equity for health equity: a rights-based approach to the control of Lassa fever in post-war Sierra Leone. BMC Int Health Hum Rights.

[28] Keenlyside RA, McCormick JB, Webb PA, Smith E, Elliot L, Johnson KM (1983). Case-control study of *Mastomy natalensis* and humans in Lassa virus-infected households in Sierra Leone. Am J Trop Med Hyg.

[29] Yalley-Ogunro JE, Frame JD, Hanson AP (1984). Endemic Lassa fever in Liberia. VI. Village serological surveys for evidence of Lassa virus activity in Lofa County, Liberia. Trans R Soc Trop Med Hyg.

[30] Leone Statistics Sierra, Thomas AC (2010). Population profile of Bo district and Bo town 2004 Census Publication Series: Number 4. Population and Housing Census of Sierra Leone.

[31] Leone Statistics Sierra, Thomas AC (2007). Population profile of Sierra Leone 2004 Census Publication Series: Number 4. Population and Housing Census of Sierra Leone.

[32] WHO (2008). Module 7: The EPI coverage survey. Training for mid-level managers (MLM)—WHO/IVB/08.07.

[33] Mills JN, Yates TL, Childs J, Parmenter RR, Ksiazek TG, Rollin PE, Peters CJ (1995). Guidelines for working with rodents potentially infected with hantavirus. J Mammal.

[34] Fichet-Calvet E, Johnson N (2014). Lassa fever: a rodent-human interaction. The Role of Animals in Emerging Viral Diseases.

[35] Lecompte E, Brouat C, Duplantier JM, Galan M, Granjon L, Loiseau A, Mouline K, Cosson JF (2005). Molecular identification of four cryptic species of *Mastomys* (Rodentia, Murinae). Biochem Syst Ecol.

[36] (2002). Lassa Fever KAP survey——Report.

[37] Masi E, Pino FA, Santos Md GS, Genehr L, Albuquerque JOM, Bancher AM, Alves JCM (2010). Socioeconomic and environmental risk factors for urban rodent infestation in Sao Paulo, Brazil. J Pest Sci.

[38] Promkerd P, Khoprasert Y, Virathavone P, Thoummabouth M, Sirisak O, Jakel T (2008). Factors explaining the abundance of rodents in the city of Luang Prabang, Lao PDR, as revealed by field and household surveys. Integr Zool.

[39] McCormick JB, Webb PA, Krebs JW, Johnson KM, Smith ES (1987). A prospective study of the epidemiology and ecology of Lassa fever. J Infect Dis.

[40] Sagripanti JL, Lytle CD (2011). Sensitivity to ultraviolet radiation of Lassa, vaccinia, and Ebola viruses dried on surfaces. Arch Virol.

[41] Monath TP (1975). Lassa fever: review of epidemiology and epizootiology. Bull World Health Organ.

[42] Borremans B, Vossen R, Becker-Ziaja B, Gryseels S, Hughes N, Van Gestel M, Van Houtte N, Gunther S, Leirs H (2015). Shedding dynamics of Morogoro virus, an African arenavirus closely related to Lassa virus, in its natural reservoir host *Mastomys natalensis*. Sci Rep.

[43] Whisson DA, Quinn JH, Collins KC (2007). Home range and movements of roof rats (*Rattus rattus*) in an old-growth Riparian forest, California. J Mammal.

[44] Foster S, King C, Patty B, Miller S (2011). Tree-climbing capabilities of Norway and ship rats. NZ J Zool.

[45] Feng AYT, Himsworth CG (2013). The secret life of the city rat: a review of the ecology of urban Norway and black rats (*Rattus norvegicus* and *Rattus rattus*). Urban Ecosyst.

[46] Fichet-Calvet E, Koulemou K, Koivogui L, Soropogui B, Sylla O, Lecompte E, Daffis S, Kouadio A, Kouassi Kan S, Akoua-Koffi C, Denys C, ter Meulen J (2005). Spatial distribution of commensal rodents in regions with high and low Lassa fever prevalence in Guinea. Belg J Zool.

[47] Fichet-Calvet E, Lecompte E, Koivogui L, Daffis S, ter Meulen J (2008). Reproductive characteristics of *Mastomys natalensis* and Lassa virus prevalence in Guinea, West Africa. Vector Borne Zoonotic Dis.

[48] Gratz NG (1997). The burden of rodent-borne diseases in Africa south of the Sahara. Belg J Zool.

[49] Han BA, Schmidt JP, Bowden SE, Drake JM (2015). Rodent reservoirs of future zoonotic diseases. Proc Natl Acad Sci USA.

[50] Wolfe ND, Dunavan CP, Diamond J (2007). Origins of major human infectious diseases. Nature.

[51] Eisen RJ, Enscore RE, Atiku LA, Zielinski-Gutierrez E, Mpanga JT, Kajik E, Andama V, Mungujakisa C, Tibo E, MacMillan K, Borchert JN, Gage KL (2013). Evidence that rodent control strategies ought to be improved to enhance food security and reduce the risk of rodent-borne illnesses within subsistence farming villages in the plague-endemic West Nile region, Uganda. Int J Pest Manage.

[52] Coeurdassier M, Riols R, Decors A, Mionnet A, David F, Quintaine T, Truchetet D, Scheifler R, Giraudoux P (2014). Unintentional wildlife poisoning and proposals for sustainable management of rodents. Conserv Biol.

[53] Brown PR, Yee N, Singleton GR, Kenney AJ, Htwe NM, Myint M, Aye T (2008). Farmers' knowledge, attitudes, and practices for rodent management in Myanmar. Int J Pest Manage.

[54] World Bank (2014). World Development Indicators: Sierra Leone.

[55] Fichet-Calvet E, Olschlager S, Strecker T, Koivogui L, Becker-Ziaja B, Camara AB, Soropogui B, Magassouba N, Gunther S (2016). Spatial and temporal evolution of Lassa virus in the natural host population in Upper Guinea. Sci Rep.

[56] Glass GE, Johnson JS, Hodenbach GA, Disalvo CLJ, Peters CJ, Childs JE, Mills JN (1997). Experimental evaluation of rodent exclusion methods to reduce hantavirus transmission to humans in rural housing. Am J Trop Med Hyg.

[57] Hopkins AS, Whitetail-Eagle J, Corneli AL, Person B, Ettestad PJ, Dimenna M, Norstog J, Creswell J, Khan AS, Olson JG, Cavallaro KF, Bryan RT, Cheek JE, Begay B, Hoddenbach GA, Ksiazek TG, Mills JN (2002). Experimental evaluation of rodent exclusion methods to reduce hantavirus transmission to residents in a native American Community in New Mexico. Vector Borne Zoonotic Dis.

